# The Role of Gut Microbiota in Thromboangiitis Obliterans: Cohort and Mendelian Randomization Study

**DOI:** 10.3390/biomedicines12071459

**Published:** 2024-07-01

**Authors:** Chang Sheng, Weihua Huang, Mingmei Liao, Pu Yang

**Affiliations:** 1 Department of Vascular Surgery, Xiangya Hospital, Central South University, Changsha 410008, China; 2 Department of Clinical Pharmacology, Hunan Key Laboratory of Pharmacogenetics, Xiangya Hospital, Central South University, Changsha 410078, China; 3 Institute of Clinical Pharmacology, Hunan Key Laboratory, Pharmacogenetics Xiangya Hospital, Central South University, Changsha 410078, China; 4 National Clinical Research Center for Geriatric Disorders, School of Pharmacy, Hunan University of Chinese Medicine, Changsha 410128, China; 5 National Health Commission Key Laboratory of Nanobiological Technology, Xiangya Hospital, Central South University, Changsha 410008, China; 6 National Clinical Research Center for Geriatric Disorders, Xiangya Hospital, Central South University, Changsha 410008, China

**Keywords:** mendelian randomization, thromboangiitis obliterans, gut microbiota, case–control study, causal correlation

## Abstract

Background and aims: Thromboangiitis obliterans (TAO), also known as Buerger’s disease, is a rare vasculitis. Observational epidemiology studies have suggested a relationship between the gut microbiota and TAO. However, due to confounding factors and reverse causality, the causal relationship remains unclear. Based on the assumption of their association, this study sought specific gut microbiota causally linked to TAO. Methods: The case–control study was conducted at the Xiangya Hospital of Central South University from November 2022 to January 2023 including twelve TAO patients and nine healthy controls. We conducted a Mendelian randomization (MR) study using summary statistics from a genome-wide association study (GWAS) of gut microbiota and TAO. Considering the scale and accessibility of the data, the MiBioGen consortium served as the exposure, whereas the FinnGen consortium GWAS study served as the outcome. Finally, we compared the results of the MR with those of the case–control studies. Results: The inverse variance weighted (IVW) (OR = 0.119, 95% CI: 0.021–0.688, *p* = 0.017) and maximum likelihood (ML) (OR = 0.121, 95% CI: 0.020–0.742, *p* = 0.022) estimates suggest that *Ruminiclostridium 5* has a suggestive protective effect on TAO while the IVW (OR = 5.383, 95% CI: 1.128–25.693, *p* = 0.035) and ML (OR = 5.658, 95% CI: 1.142–28.021, *p* = 0.034) estimates suggest that *Eubacterium* (*xylanophilum group*) has a suggestive risk effect on TAO, and the ML (OR = 0.055, 95% CI: 0.004–0.755, *p* = 0.030) estimates suggest that *Lachnospira* has a suggestive protective effect on TAO. No significant heterogeneity of instrumental variables or horizontal pleiotropy was found. The results of the case–control study showed that the TAO had a lower relative abundance of *Ruminiclostridium 5* (*p* = 0.015) and *Lachnospira* (*p* = 0.048), and a higher relative abundance of *Eubacterium* (*xylanophilum group*) (*p* = 0.029) than the healthy controls. These results were consistent with the MR analysis. Conclusions: Our study demonstrates that *Ruminiclostridium 5*, *Lachnospira*, and *Eubacterium* (*xylanophilum group*) are causally related to TAO, suggesting their potential significance for the prevention and treatment of TAO.

## 1. Introduction

Thromboangiitis obliterans (TAO) is a chronic inflammatory disease that primarily affects small- and medium-sized arteries and veins [1], characterized by the infiltration of inflammatory cells and thrombosis. The risk factors for TAO include smoking, periodontal disease, and genetic factors [2]. However, the exact cause of the disease remains unknown, and both the underdiagnosis and misdiagnosis of TAO continue to occur at high rates [2,3]. Although surgery is the primary treatment, long-term vascular patency rates are poor, and additional interventions are often necessary. Patients frequently suffer from symptoms such as active ulcers and resting pain, leading to a poor quality of life [4,5].

The human gastrointestinal microbiota plays a crucial role in immunological functions and metabolic processes including host immunity, food digestion, intestinal endocrine function, and intestinal permeability [6]. These microbial metabolites can enter the peripheral circulation through the portal vein [7] or are easily absorbed by the gut mucosa [8] where they can act as substrates or signaling molecules in organs. Microbiota can also exacerbate systemic vascular inflammation and generate bacterial by-products through metabolism-independent mechanisms [9]. The gut microbiota is a crucial underlying player during sepsis pathogenesis [10]. While research has paid more attention to the relationship between the gut microbiota and cardiovascular disease [11,12,13,14,15], research into the gut microbiota and TAO is limited. Studies have found that almost two-thirds of TAO patients suffer from severe periodontal disease [16]. Anaerobic DNA fragments such as *Campylobacter rectus*, *Porphyromonas gingivalis*, and *Prevotella* have been discovered in the diseased arteries of TAO patients [16]. However, the origins of these pathogenic bacteria need further exploration. In observational studies, the association between gut microbiota and TAO is susceptible to confounding factors such as age, environment, dietary patterns, and lifestyle [17,18,19].

Mendelian randomization (MR) offers a new approach to investigate the causal link between the gut microbiota and TAO. The principle of gut microbiota genome-wide association studies (GWASs) involves sequencing the genotype and microbial composition of large-scale samples, followed by statistical analysis of their correlation to determine the genetic basis of specific microbial populations [20]. Twin studies have shown that the abundance of various bacterial taxa has an average heritability of 20% [21,22], supporting the notion that genetic factors play a significant role in shaping the gut microbiota composition. MR employs genetic variants as instrumental variables of exposure to estimate the causal relationship between exposure and outcome [23], as genotypes are randomly allocated from the parents to offspring, lending credence to a causal sequence. MR has been extensively used to explore the causal association between the gut microbiota and several diseases including metabolic diseases [24], autoimmune diseases [25], and rheumatoid arthritis [26]. In this study, we conducted a two-sample MR analysis using GWAS summary statistics from the MiBioGen and FinnGen consortiums to assess the causal link between the gut microbiota and TAO. Additionally, we conducted a case–control study to investigate alterations in microbial abundance among TAO patients, confirming our MR results.

## 2. Materials and Methods 

### 2.1. Assumptions of MR

**Figure 1:**
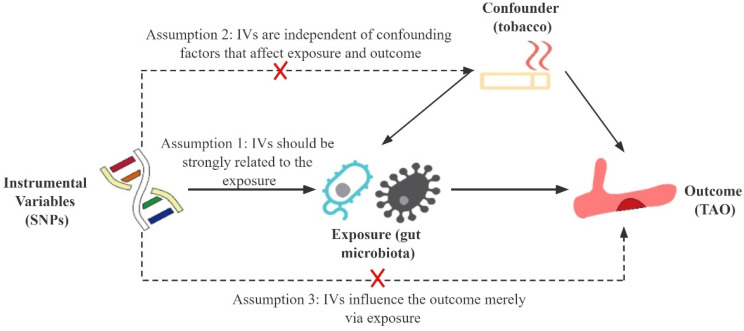
Schematic representation of this Mendelian randomization study. SNPs, single nucleotide polymorphisms; IVs, instrumental variables; TAO, thromboangiitis obliterans.

This study followed the MR reporting guidelines [27]. The MR approach is based on three primary assumptions (Figure 1) [28]. First, the instrumental variables (IVs) in the form of SNPs must exhibit a substantial correlation with the gut microbiota and achieve genome-wide significance (Assumption 1). Second, the IVs should be independent of confounding factors (Assumption 2). Third, the IVs should only affect TAO via the gut microbiota pathway while precluding other pathways (Assumption 3).

### 2.2. Selection of Instrumental Variables

To identify the genetic variants associated with gut microbiota, the large meta-analysis of GWAS conducted by the MiBioGen consortium was utilized (https://mibiogen.gcc.rug.nl/, accessed on 15 February 2023) [20]. The study included 18,340 individuals from 24 cohorts, with the majority being of European descent (n = 13,266). They targeted variable regions V4, V3–V4, and V1–V2 of the 16S rRNA gene to profile the microbial composition and employed direct taxonomic binning to conduct the taxonomic classification. In this study, the lowest taxonomic level considered was the genus, and 131 genera with a mean abundance higher than 1% were identified including 12 unknown genera [20].

The selection criteria for the IVs were as follows. (1) The locus-wide significance threshold is more stringent compared to the genome-wide significance threshold. SNPs associated with each genus at the locus-wide significance threshold (*p* < 1.0 × 10^−5^) were chosen as potential IVs [24]. (2)The PLINK clumping method was utilized with a more stringent clumping threshold (*r*^2^ < 0.001, *kb* = 10,000) to prune SNPs in LD within a given window to account for residual linkage disequilibrium (LD) of the genetic variants [29]. (3) The *F*-statistic was calculated to evaluate the strength of the IVs using the formula $F=\frac{R^{2}\times \left(N-1-K\right)}{\left(1-R^{2}\right)\times K}$. If the corresponding *F*-statistic was >10, no significant weak instrumental bias was assumed [30]. (4) Tobacco exposure is critical to TAO initiation, maintenance, and progression [1] and may impact the MR findings as a confounding factor. Therefore, we examined the PhenoScanner [31] (http://www.phenoscanner.medschl.cam.ac.uk/, accessed on 5 March 2023) database, which contains publicly available information on a large number of genetic variants, to identify and exclude IVs directly linked to tobacco exposure or factors affecting the gut microbiota to address the MR assumptions. (5) SNPs with a palindromic structure were automatically eliminated during the analysis.

### 2.3. Data Sources of Outcomes

We obtained the summary statistics for TAO from the R7 release data of the FinnGen consortium [32] (https://r7.finngen.fi/, accessed on 10 February 2023), which included a total of 16,383,294 variables from Finnish biobanks. The GWAS comprised 288,723 participants including 85 cases (53 males and 32 females) and 288,638 controls. To adjust for potential confounding factors, we controlled for sex, age, first 10 principal components, and genotyping batch during the analysis [32]. To ensure uniform reference alleles, the exposure and outcome data were combined and standardized. We then oriented the summary effect estimates in the same direction as those in the 1000 genomes reference dataset [33].

### 2.4. Case–Control Study

The case–control study was conducted at Xiangya Hospital of Central South University between November 2022 and January 2023. Individuals meeting the following inclusion criteria for TAO were as follows [1]: (1) young and middle-aged individuals with a smoking history; (2) varying degrees of ischemic symptoms in the affected limb; (3) decreased or absent pulsation of the dorsalis pedis artery and/or posterior tibial artery on the affected side; (4) TAO suggested by CT angiography. We then selected a certain number of varicose vein patients as the control group and conducted physical examinations during the same period. Fecal specimens were collected after the diagnosis of TAO but before the treatment. To ensure the representativeness of fecal specimens, we controlled for confounding factors that can alter the gut microbiota community. The exclusion criteria were as follows [18,34]: (1) history of antibiotic, probiotic, hormone, and gastrointestinal disease-related drug use, alcohol and drug abuse within the last month; (2) metabolic diseases such as diabetes, rheumatoid arthritis, severe organ damage, tumors, infectious diseases, food intolerance, and a history of inflammatory bowel disease; (3) previous long-term or recent history of bloating, diarrhea, gastrointestinal discomfort such as constipation; (4) short-term change in diet structure; (5) definite autoimmune diseases, hypercoagulable blood, popliteal artery trapping syndrome, and patients with emboli of proximal origin; (6) non-Han Chinese. Demographic information was recorded for all of the study participants.

In order to minimize the potential impact of collecting samples from different fecal locations, we collected the middle feces from all participants as samples. The QIAGEN Fecal DNA Extraction Kit (QIAamp PowerFecal Pro DNA Kit) (Cat NO. 51804) was utilized to extract fecal DNA in this study. The extracted fecal DNA was qualified and sent to Nuohe Zhiyuan Company (Beijing, China) for 16s rRNA amplification and sequencing. Before sequencing, a quantitative analysis was carried out, requiring that the DNA concentration of the sample to be tested be ≥ 10 ng/μL, with a mass ≥ 500 ng. DNA amplification was followed by splicing to construct a library, which was then quantified and sequenced using a computer. After off-board data analysis, MEGAHIT assembly, QUEST evaluation, Prokka gene annotation, and MetaPhlAn2 species abundance analysis were performed. The human study was approved by the Institutional Review Board of Xiangya Hospital (Approval Number: 2022111192).

### 2.5. MR Analysis and Statistical Analysis

This study utilized multiple methods including the inverse variance weighted (IVW), maximum likelihood (ML), MR-Egger regression, weighted median, weighted model, and MR-PRESSO to examine the possible causal association between the gut microbiota and TAO. The IVW method employed a meta-analysis approach that combined with the Wald estimates for each SNP to obtain an overall effect estimate of the gut microbiota on TAO. The results are unbiased if horizontal pleiotropy is absent [35]. Similar to IVW, the ML method assumes no heterogeneity or horizontal pleiotropy. If these hypotheses are met, the results will be unbiased, and the standard errors will be smaller than those obtained with IVW [36]. MR-Egger regression is based on the instrument strength independent of the direct effect (InSIDE) assumption, which enables the assessment of pleiotropy by examining the intercept term. If the intercept term equals zero, it suggests that horizontal pleiotropy does not exist, and the result of the MR-Egger regression is consistent with that of IVW [37]. The weighted median method allows for the correct estimation of causal association even when up to 50% of the instrumental variables are invalid [38]. If the InSIDE assumption fails, the weighted model estimate has been shown to have greater power to detect a causal effect, less bias, and lower type I error rates than MR-Egger regression [38]. The MR-PRESSO analysis detects and attempts to reduce horizontal pleiotropy by removing significant outliers. However, the MR-PRESSO outlier test requires that at least 50% of genetic variants be valid instruments and relies on InSIDE assumptions [39].

To quantify the heterogeneity of IVs, Cochran’s IVW *Q* statistics were conducted. The Cochran *Q* test was used to test for heterogeneity, with a *p*-value < 0.05 indicating significantly heterogeneous. Furthermore, a “leave-one-out” analysis was carried out by excluding each instrumental SNP in turn to identify potential heterogeneous SNPs. Directional pleiotropy was assessed through visual inspection of SNP scatter plots and funnel plots, and formally tested using the Egger intercept test.

The power of the MR estimates was calculated using an online calculator tool (https://sb452.shinyapps.io/power/, accessed on 13 March 2023) provided by Stephen Burgess [40]. With Bonferroni correction, the adjusted multiple comparisons *p*-value < 0.00042 (0.05/119) was considered to be statistically significant. A suggestive association between the genera of gut microbiota and TAO was defined as a *p*-value > 0.00042, but <0.05. All statistical data analyses were conducted with R software, version 4.2.1 (http://www.r-project.org, accessed on 3 January 2023), with the TwoSampleMR (version 0.5.6) [41] and MRPRESSO packages (version 1.0) [39]. We also performed a reverse MR analysis on the bacteria that showed causality in the positive MR analysis for TAO to exclude potential reverse associations. The methodologies and configurations employed were analogous to those used in the forward MR analysis.

## 3. Results

### 3.1. Genetic Associations between Gut Microbiota and TAO 

**Figure 2:**
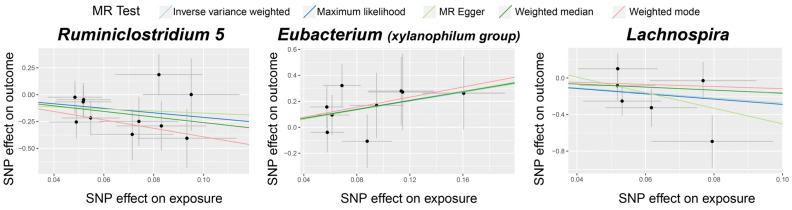
Scatter plots for the causal association between gut microbiota and TAO. The dots in the graph represent SNPs.

Supplementary Table S1 details the selected IVs. As presented in Table 1 and Figure 2, we identified three bacterial genera, namely *Ruminiclostridium 5*, *Eubacterium* (*xylanophilum group*), and *Lachnospira*, as linked to TAO. The IVW (OR = 0.119, 95% CI: 0.021–0.688, *p* = 0.017) and ML (OR = 0.121, 95% CI: 0.020–0.742, *p* = 0.022) estimates indicated a suggestive protective effect of *Ruminiclostridium 5* on TAO. The IVW (OR = 5.383, 95% CI: 1.128–25.693, *p* = 0.035) and ML (OR = 5.658, 95% CI: 1.142–28.021, *p* = 0.034) estimates suggested a suggestive risk effect of *Eubacterium* (*xylanophilum group*) on TAO. Although the IVW estimates did not support the causal association of *Lachnospira* on TAO, the ML estimates suggested a possible relationship (OR = 0.055, 95% CI: 0.004–0.755, *p* = 0.030). The reverse MR did not yield positive results. *F*-statistics of the IVs for these three causal associations ranged from 12.03 to 26.97, which eliminated the bias of weak IVs. The Cochran’s IVW *Q* test results showed no significant heterogeneity among these IVs (Supplementary Table S2).

**Figure 3:**
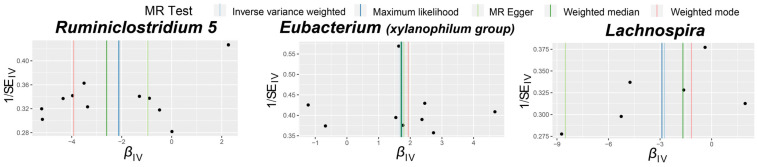
Funnel plots for the causal association between gut microbiota and TAO. The dots in the graph represent SNPs.

**Figure 4:**
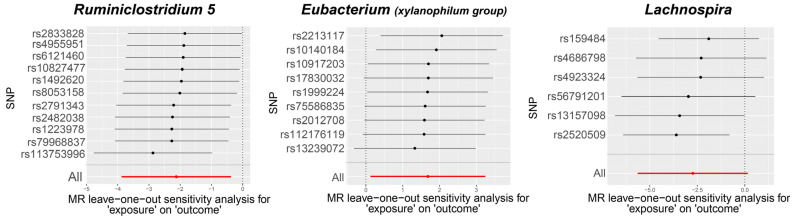
Leave-one-out plots for the causal association between gut microbiota and TAO.

Visual inspection of the funnel plots (Figure 3) and leave-one-out plots (Figure 4) revealed potential outliers for the IVs of *Eubacterium* (*xylanophilum group*) and *Lachnospira*. However, no instrumental variables significantly influenced the overall results. The MR-PRESSO analysis did not identify any significant outliers (Global test *p* > 0.05, Supplementary Table S3). The MR-Egger regression intercept analysis (Supplementary Table S4) did not detect significant horizontal pleiotropy.

### 3.2. Case–Control Study

A case–control study was conducted, and the baseline demographic and clinicopathologic features were compared and presented in Table 2.

**Figure 5:**
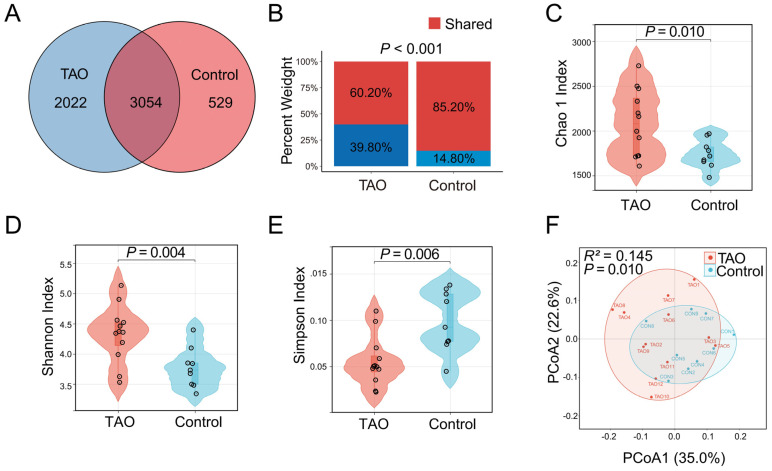
Differences in gut microbiome composition and distribution in the TAO patients and controls. (**A**) The numbers of OTUs in the TAO patients, controls, and shared OTUs in both groups in a scaled Venn diagram. (**B**) Total abundance of OTUs in the TAO patients and controls. α−Diversity was described by the Chao1 index (**C**), Shannon index (**D**), and Simpson (**E**). β−Diversity was described by principal coordinates analysis (PCoA) based on the weighted UniFrac distance metric (**F**).

A total of 5076 operational taxonomic units (OTUs) were detected in the TAO patients, while 3583 OTUs belonged to the gut microbiota in the controls (Figure 5A). Among these, 3054 OTUs constituted common microbiota, which showed lower relative abundance in TAO patients (Figure 5B). α-Diversity and β-diversity were used to describe the microbial composition and distribution. Sequences with ≥ 100% similarity were classified as the same feature. The Chao1 index, Shannon index, and Simpson index to were used to describe the α-diversity further. The violin plot of the Chao1 index revealed statistically significant differences between the TAO patients and controls (*p* = 0.029) (Figure 5C). Additionally, the violin plots of the Shannon index suggested statistical significance differences between the TAO patients and controls (*p* = 0.004), while the Simpson index suggested statistical significance among the TAO patients versus controls (*p* = 0.006) (Figure 5D,E). Principal coordinates analysis (PCoA) based on the weighted UniFrac distance to assess β-diversity indicated that the two groups differed in significance in the gut microbiological composition (*R^2^* = 0.145, *p* = 0.010) (Figure 5F).

**Figure 6:**
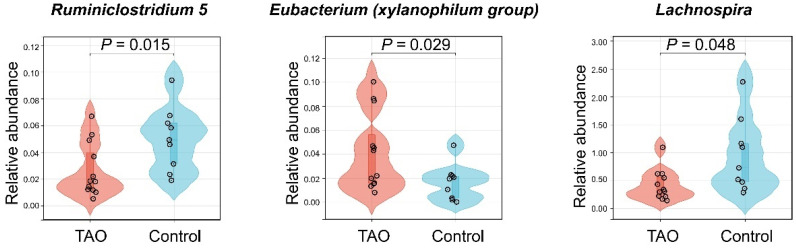
Violin plots to validate MR. Comparative relative abundance of *Ruminiclostridium 5*, *Eubacterium* (*xylanophilum group*), and *Lachnospira* in TAO and controls.

To validate the findings of the MR, the relative abundance of *Ruminiclostridium 5*, *Eubacterium* (*xylanophilum group*), and *Lachnospira* was compared between the TAO patients and controls using violin plots. TAO patients had a lower relative abundance of *Ruminiclostridium 5* (*p* = 0.015) and *Lachnospira* (*p* = 0.048), but a higher relative abundance of *Eubacterium* (*xylanophilum group*) (*p* = 0.029) than the controls (Figure 6). These results were consistent with the MR findings, indicating that the gut microbiota may have risky or protective characteristics for TAO. These results are consistent with the conclusions drawn from the MR. To predict microbial functional, we utilized the Phylogenetic Investigation of Communities by Reconstruction of Unobserved States (PICRUSt) and Clusters of Orthologous Genes (COG) database [42,43]. COG entries meeting a *p*-value of less than 0.05 and |log_2_ ^(fold change (FC))^| > 1 were defined as differential abundant proteins. We discovered that proteins such as cAMP phosphodiesterase, ABC-type sulfate transport system (periplasmic component), DNA-binding transcriptional regulator (MltR family), and DNA-binding transcriptional regulator SgrR of sgrS sRNA (contains a MarR-type HTH domain and a periplasmic-type solute-binding domain) differed between the TAO patients and controls (Supplementary Figure S1). These components are involved in regulating intracellular signaling and gene expression, which are associated with vascular inflammatory responses [44,45].

## 4. Discussion

### 4.1. Principal Findings

In our investigation, we employed summary statistics from the largest GWAS meta-analysis of gut microbiota conducted by the MiBioGen consortium as well as summary statistics of TAO from the FinnGen consortium R7 release data to conduct a two-sample MR and an analysis of human fecal specimens. Our objective was to evaluate the causal relationship between the gut microbiota and TAO. To our knowledge, this is the first to apply the concept to investigate the causality between the gut microbiota and TAO. Our findings revealed that *Ruminiclostridium 5* and *Lachnospira* had protective effects, while *Eubacterium* (*xylanophilum group*) had risk effects on TAO. 

### 4.2. Comparison with Other Studies and Possible Mechanisms

The gut microbiome is a vast collection of microorganisms in the human digestive tract that can influence the physiological functions of the gastrointestinal tract [46] and interact with almost all human cells [47]. It plays important physiological functions in the human organism including bile acid metabolism [46], synthesis of essential vitamins [48], and regulation of the immune system [49]. Currently, exploring the gut–vascular axis provides a unique insight into cardiovascular disease [13]. *Ruminiclostridium 5*, known as a probiotic [50,51], has been reported to have a protective effect on cardiovascular health [52]. Furthermore, correlation analysis showed that *Ruminiclostridium 5* was significantly related to several intestinal permeability characteristics such as fecal and serum lipopolysaccharide (LPS) and serum proinflammatory cytokines [52]. Dysregulation of the gut microbiome can lead to high levels of microbiota-associated molecular patterns (MAMPs), resulting in the induction of hepatocarcinogenesis by diethylnitrosamine (DEN) and carbon tetrachloride (CCl4) binding to LPS and their receptors, Toll-like receptor 4 (TLR4) [53]. Studies have also demonstrated that the oxidative stress levels in TAO patients are significantly higher than their antioxidant capacity [54]. The interaction of LPS with TLR4 can activate the NF-κB pathway and three MAPK pathways, producing inflammatory mediators such as IL-6 and IL-8 [55,56]. Another study has shown that TLR4 levels are significantly higher in patients with acute TAO than in patients with quiescent TAO [57]. Vitamin D supplementation has been found to increase the abundance of *Lachnospira* in human feces [58]. Patients with atrial fibrillation shown an increase in intestinal symbiotic bacteria *Lachnospira* after catheter ablation, suggesting a beneficial effect of gut microbiota. The immune system and inflammation are proposed to play a central role in TAO pathogenesis [59]. Further clarification is needed to determine whether gut microbiota is involved in the development of TAO through LPS/TLR4.

Considerable knowledge has already been accumulated regarding the role of intestinal microorganisms in immune-inflammatory diseases. *Lachnospira* has been identified as significantly downregulated in many immune-inflammatory diseases [60]. When a drug was used to treat acute coronary syndrome, the levels of the intestinal metabolite ethanolamine (EA) were significantly altered, with *Eubacterium* (*xylanophilum group*) significantly correlated with the levels of EA [61]. The metabolites or metabolomes of the gut microbiome can partially predict the etiology and progression of TAO. Certain short-chain-fatty acids (SCFAs) produced by probiotics are the primary energy sources of intestinal epithelial cells and participate in cell proliferation and differentiation, thereby maintaining cell homeostasis through anti-inflammatory and antioxidant effects [62,63]. Therefore, probiotics and SCFAs may help TAO patients maintain intestinal barrier function and prevent systemic or vascular inflammation caused by the migration of pathogenic bacteria to reduce the risk of TAO. With a focus on the gut microbiota involved in this study, further exploration is needed to understand the complex interplay between the gut microbiota and TAO. 

### 4.3. Strengths and Limitations

This study has several strengths. First, the MR design reduced the confounding bias and reverse causation, which are not well-controlled in traditional observational epidemiology. The consistency of effects across the MR models and sensitivity analysis enhanced the robustness of the results. Second, we established a population cohort and confirmed the MR results well, with differences in gut microbiota abundance. Third, we obtained genetic variants of gut microbiota from the largest available GWAS meta-analysis, ensuring strong instruments. Finally, it is worth mentioning that the three major assumptions of MR were well-adhered to. However, with the increase in future research, the IVs may undergo changes, potentially affecting the main results. 

Several limitations should be considered when interpreting the results. As we used summary statistics rather than raw data, distinguishing TAO with varying degrees of ischemia or gender or exploring quantitative relationships was not possible. The lowest taxonomic level available in the exposure dataset was the genus, restricting our ability to explore the causal association between the gut microbiota and TAO at the species level. Given the absence of significant horizontal pleiotropy and the effectiveness of instrumental variables, IVW and ML were appropriate methods in our study. Although these results were not highly significant, they reflect the real scenario. Furthermore, currently, the data have their own limitations, and methods with a higher resolution for species identification are worth considering, although such methods require more resources. To detect horizontal pleiotropy and conduct sensitivity analysis, more genetic variations need to be included as IVs; therefore, the SNPs used in the analysis did not reach the traditional GWAS significance threshold (*p* < 5 × 10^−8^) [64,65]. Although most participants in the GWAS meta-analysis for gut microbiota data were of European descent, interference from population stratification may still exist, and the findings may not be entirely applicable to subjects of non-European descent. The inconsistency in the ancestry of the exposed, outcome, and validation dataset populations warrants a cautious interpretation of the results. Additionally, the relatively low sample size of the analyses may limit the statistical power to detect genetic associations. The lower sample size in the case–control study also suggests that the current conclusions need to be interpreted with caution. Lifestyle factors such as smoking, diet, and physical activity may play a role in the current associations, which require further analysis. Large-scale studies in diverse populations are necessary to establish the consistency of the results across different ethnic groups.

## 5. Conclusions

In conclusion, this study involved both two-sample MR and case–control analyses and demonstrated a causal link between *Ruminiclostridium 5*, *Lachnospira*, and *Eubacterium* (*xylanophilum group*) with TAO. These gut microbes could serve as potential markers or a therapeutic target for TAO. Additional randomized controlled trials are necessary to clarify the impact of probiotics on TAO and its specific mechanism.

## Figures and Tables

**Table 1:** MR estimates for the association between gut microbiota and TAO.

Bacterial Taxa (Exposure)	MR Method	No. of SNP	*F*-Statistic	Power	OR	95% CI	*p*-Value
*Ruminiclostridium 5*	IVW	11	12.03	0.13	0.119	0.021–0.688	0.017
	ML	11			0.121	0.020–0.742	0.022
	MR-Egger	11			0.385	0.000–572.786	0.804
	Weighted median	11			0.074	0.007–0.752	0.028
	Weighted mode	11			0.020	0.000–1.549	0.108
*Eubacterium* (*xylanophilum group*)	IVW	9	24.67	1.00	5.383	1.128–25.693	0.035
	ML	9			5.658	1.142–28.021	0.034
	MR-Egger	9			6.043	0.055–660.248	0.477
	Weighted median	9			5.545	0.718–42.852	0.101
	Weighted mode	9			6.942	0.364–132.271	0.234
*Lachnospira*	IVW	6	26.97	0.12	0.064	0.003–1.176	0.064
	ML	6			0.055	0.004–0.755	0.030
	MR-Egger	6			0.000	0.000–15,532.199	0.410
	Weighted median	6			0.189	0.006–5.775	0.339
	Weighted mode	6			0.309	0.004–25.026	0.623

MR, Mendelian randomization; TAO, thromboangiitis obliterans; SNP, single nucleotide polymorphism; OR, odds ratio; CI, confidence interval; IVW, inverse variance weighted; ML, maximum likelihood.

**Table 2:** Demographic and clinicopathologic features of the participants in the case–control study.

	TAO (n = 12)	Controls (n = 9)
Age, median (IQR), y	39 (32.25, 44.25)	42 (32.00, 45.50)
Gender, No. (%)		
Male	12 (100)	9 (100)
Female	0 (0)	0 (0)
Smoking history, No. (%)		
Yes	12 (100)	7 (78)
No	0 (0)	2 (22)
Intermittent claudication, No. (%)		
Yes	12 (100)	0 (0)
No	0 (0)	0 (0)
Resting pain, No. (%)		
Yes	9 (75)	0 (0)
No	3 (25)	0 (0)
Ulcer or gangrene, No. (%)		
Yes	10 (83)	0 (0)
No	2 (17)	0 (0)
Migratory superficial phlebitis, No. (%)		
Yes	6 (50)	0 (0)
No	6 (50)	0 (0)
Reynolds phenomenon, No. (%)		
Yes	4 (33)	0 (0)
No	8 (67)	0 (0)

IQR, interquartile range.

## Data Availability

All data and materials supporting the findings of this work are available from the corresponding author upon reasonable request. The data are not publicly available due to privacy concerns and restrictions imposed by the ethical review board.

## Supplementary Materials

The following supporting information can be downloaded at: https://www.mdpi.com/article/10.3390/biomedicines12071459/s1, Table S1. Instrumental variables used in MR analysis of the association between gut microbiota and TAO. Table S2. The heterogeneity of gut microbiota instrumental variables. Table S3. MR-PRESSO analysis for the association between gut microbiota and TAO. Table S4. Directional horizontal pleiotropy assessed by intercept term in MR Egger regression of the association between TAO and gut microbiota. Figure S1. Functional alterations in gut microbiota in TAO patients.

## Abbreviations

TAO	Thromboangiitis obliterans
MR	Mendelian randomization
GWAS	Genome-wide association study
SNPs	Single nucleotide polymorphisms
IVs	Instrumental variables
IVW	Inverse-variance weighted
ML	Maximum likelihood
LPS	Lipopolysaccharide
mbQTL	Microbiota quantitative trait loci
LD	Linkage disequilibrium
ICD-10	International Classification of Diseases 10th version
InSIDE	Instrument strength independent of direct effect
OR	Odds ratio
CI	Confidence interval
PCoA	Principal coordinates analysis
MAMPs	Microbiota-associated molecular patterns
TLR4	Toll-like receptor 4
EA	Metabolite ethanolamine
SCFAs	Short-chain-fatty acids
