# Supplementary material for: The Role of Gut Microbiota in Thromboangiitis Obliterans: Cohort and Mendelian Randomization Study
Source: Biomedicines. 2024 Jul 1;12(7):1459. doi: 10.3390/biomedicines12071459 (PMC11274368; doi:10.3390/biomedicines12071459)
Supplement: Supplementary file 1 [file biomedicines-12-01459-s001.zip › biomedicines-3016654-supplementary.pdf]

Table S1. Instrumental variables used in MR analysis of the association between gut microbiota and TAO.

Exposure	SNP	Effect allele	Other allele	MAF	Exposure			Outcome		
					Beta	SE	*P*-value	Beta	SE	*P*-value
Ruminiclostridium 5	rs79968837	A	G	0.051	-0.095	0.019	1.15E-06	-0.001	0.337	0.998
Ruminiclostridium 5	rs2482038	C	A	0.403	0.052	0.011	1.70E-06	-0.046	0.154	0.763
Ruminiclostridium 5	rs10827477	A	G	0.353	-0.055	0.012	2.19E-06	0.217	0.160	0.175
Ruminiclostridium 5	rs6121460	G	A	0.082	0.093	0.020	2.64E-06	-0.405	0.277	0.143
Ruminiclostridium 5	rs1492620	T	C	0.130	-0.083	0.018	3.53E-06	0.291	0.229	0.203
Ruminiclostridium 5	rs113753996	T	C	0.182	0.082	0.017	3.99E-06	0.186	0.192	0.333
Ruminiclostridium 5	rs2791343	T	C	0.398	0.052	0.011	5.54E-06	-0.067	0.152	0.660
Ruminiclostridium 5	rs8053158	A	G	0.122	-0.074	0.016	5.90E-06	-0.249	0.229	0.277
Ruminiclostridium 5	rs2833828	G	A	0.412	0.049	0.011	6.82E-06	-0.255	0.153	0.096
Ruminiclostridium 5	rs1223978	T	C	0.469	0.048	0.011	8.16E-06	0.024	0.152	0.877
Ruminiclostridium 5	rs4955951	A	G	0.117	-0.071	0.017	9.96E-06	0.369	0.236	0.118
Eubacterium (xylanophilum group)	rs17830032	G	A	0.081	-0.161	0.031	2.39E-07	-0.263	0.282	0.352
Eubacterium (xylanophilum group)	rs13239072	G	A	0.276	0.069	0.014	1.82E-06	0.321	0.168	0.056
Eubacterium (xylanophilum group)	rs10917203	A	C	0.394	0.061	0.013	3.15E-06	0.095	0.155	0.542
Eubacterium (xylanophilum group)	rs112176119	C	T	0.093	-0.113	0.025	3.33E-06	-0.279	0.264	0.291
Eubacterium (xylanophilum group)	rs1999224	G	T	0.098	-0.095	0.020	3.75E-06	-0.168	0.253	0.505
Eubacterium (xylanophilum group)	rs2213117	T	G	0.164	0.088	0.019	4.21E-06	-0.107	0.206	0.603
Eubacterium (xylanophilum group)	rs10140184	A	C	0.475	0.058	0.013	4.96E-06	-0.039	0.154	0.800
Eubacterium (xylanophilum group)	rs2012708	A	G	0.343	0.057	0.013	6.53E-06	-0.157	0.160	0.329
Eubacterium (xylanophilum group)	rs75586835	A	G	0.071	-0.114	0.026	9.39E-06	-0.271	0.294	0.356
Lachnospira	rs13157098	A	G	0.163	-0.077	0.016	5.99E-07	0.029	0.204	0.886
Lachnospira	rs4923324	G	A	0.161	-0.062	0.013	2.44E-06	0.325	0.207	0.117
Lachnospira	rs4686798	T	C	0.362	0.053	0.011	2.74E-06	-0.253	0.158	0.109
Lachnospira	rs56791201	T	C	0.362	0.052	0.011	2.93E-06	0.084	0.158	0.594
Lachnospira	rs159484	G	A	0.073	0.079	0.018	6.68E-06	0.694	0.286	0.015
Lachnospira	rs2520509	A	G	0.291	0.052	0.012	7.42E-06	-0.101	0.166	0.542

MR, Mendelian randomization; TAO, thromboangiitis obliterans; SNP, single nucleotide polymorphism; MAF, minor allele frequency; SE, Standard error.

Table S2. The heterogeneity of gut microbiota instrumental variables.

Exposure	Cochran's *Q*	degree of freedom	*P*-value
Ruminiclostridium 5	7.584	10	0.669
Eubacterium (xylanophilum group)	4.126	8	0.846
Lachnospira	6.868	5	0.231

Table S3. MR-PRESSO analysis for the association between gut microbiota and TAO.

Exposure	MR anlysis	Causal estimate	SD	T	*P*-value	RSS_{obs}	Global test *P*-value
Ruminiclostridium 5	MR-PRESSO	-1.464	0.736	-1.989	0.667	18.581	0.330
Eubacterium (xylanophilum group)	MR-PRESSO	0.752	0.689	1.092	0.298	12.375	0.498
Lachnospira	MR-PRESSO	-3.014	1.243	-2.426	0.051	9.403	0.373

MR, Mendelian randomization; TAO, thromboangiitis obliterans; SD, standard deviation; RSS_{obs}, observed residual sum of squares.

Table S4. Directional horizontal pleiotropy assessed by intercept term in MR Egger regression of the association between TAO and gut microbiota.

Exposure	Egger intercept	SE	*P*-value
Ruminiclostridium 5	-0.076	0.235	0.753
Eubacterium (xylanophilum group)	-0.010	0.196	0.961
Lachnospira	0.350	0.553	0.562

MR, Mendelian randomization; TAO, thromboangiitis obliterans; SE, standard error.

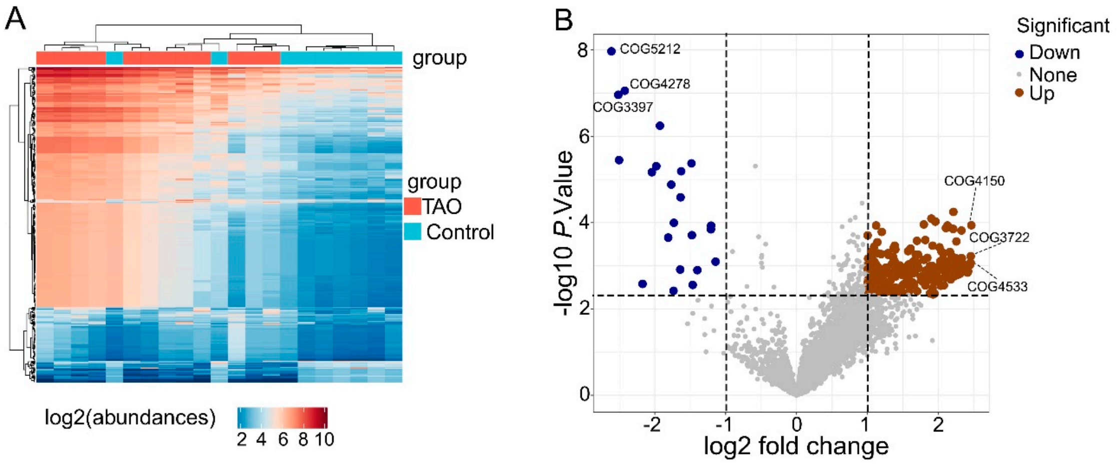

Figure S1. Functional alterations in gut microbiota in TAO patients. (A) Expression level heatmap and hierarchical clustering of COG entries between TAO patients and controls. (B) Volcano plot of COG entries between TAO patients and controls. COG5212: cAMP phosphodiesterase; COG4278: Uncharacterized protein; COG3397: Predicted carbohydrate-binding protein (contains CBM5 and CBM33 domains); COG4150: ABC-type sulfate transport system (periplasmic component); COG3722: DNA-binding transcriptional regulator (MltR family); COG4533: DNA-binding transcriptional regulator SgrR of sgrS sRNA (contains a MarR-type HTH domain and a periplasmic-type solute-binding domain).
